# Medical Opinion and Movement

**Published:** 1908-02-15

**Authors:** 


### MEDICAL OPINION AND MOVEMENT.

THE introduction of medicaments into the system by means of electrolysis, or so-called "ionic medication," has already received notice in these columns. It is largely due to Prof. Leduc, of Nantes, that the method has been developed. It has been shown to be one of considerable practical utility in a variety of conditions, and it seems probable that it will increase in popularity among the profession. It has been found a useful means of introducing iodine into sprained joints, and also for the absorption of gouty and rheumatic deposits. A pad soaked with a watery solution of iodine is placed around the joint and covered with a metal plate connected with the positive pole of the battery. In a similar way local anæsthesia can be produced with a solution of cocaine, but in this case the negative pole must be connected with the part to be affected. Rodent ulcers have been very successfully treated by electrolysis with zinc chloride solution, and good results have also been obtained in other similar superficial ulcerations. There are many technical details to be studied in regard to the strength of the current, kind of electrode and nature and strength of the solutions to be used, and there is undoubtedly a wide scope for further investigations on these lines.

PROFESSORS GERARD and LEMOINE have been investigating the properties of bile with a view to discover a substance possessing antitoxic properties against tuberculous infections. They were led to these researches by the observations of Phisalyx in 1897, who showed that bile and the biliary acids neutralise snake-venom, and that cholesterin acted as an antitoxin to snake-venom. Taking the view that the liver is one of the chief seats of defence in the body against infections and intoxications, it seemed possible that substances of medicinal value might be extracted from bile. They have now succeeded in making a preparation from bile which they term paratoxine, and for which they claim special therapeutic value in tuberculous affections. This preparation can be administered by the mouth or injected subcutaneously, and it is said to form a most efficient medicament for intralaryngeal injections in cases of tuberculous laryngitis. In pulmonary tuberculosis the general symptoms—fever, anorexia, night sweats, and general malaise—are said to undergo rapid improvement, and at the same time expectoration diminishes, the number of bacilli decrease, and the pulmonary lesions tend to heal. It is admitted that in very acute or advanced cases paratoxine is of no great value, but, on the other hand, tuberculous enteritis is said to yield to the treatment in a marked manner. Confirmation of these results by other observers will be awaited with interest.

PHYSIOLOGISTS were not slow to recognise in the $x$-rays a new means of research on the digestive processes and movements of the stomach and intestine, and our knowledge in this direction has been materially advanced by Cannon, and later by Rieder and Holzknecht, by their skiagraphic observations on animals and on man. Quite recently these observations have been confirmed and extended by Dr. A. F. Hertz at Guy's Hospital, and by combining skiagraphy with auscultation and percussion he has been able to give a very useful account of the course of the food through the alimentary canal. The food is made visible by means of the $x$-rays by mixing a meal with an ounce or more of bismuth carbonate. In this way it has been demonstrated that the stomach is functionally quite definitely divided into two parts—the fundus, or upper third, which acts as a sort of storehouse and shows little or no digestive activity, and the antrum pylori, which receives the food in doles and is subject to constant peristalsis from the commencement of a meal. When empty the fundus contains gas, but the rest of the stomach is collapsed. It appears from these observations that in the recumbent position food does not pass from the fundus to the collapsed pyloric part of the stomach till sufficient has been received to overcome the resistance due to the cohesion of the stomach walls, and it is suggested that the feeds often given to patients in bed may be too small to effect this, and consequently remain in the fundus undigested till a further feed is administered. The indication would be to give larger feeds at longer intervals. One must not, however, be too hasty to apply physiological deductions to bedside treatment. On other grounds, probably in the majority of cases, frequent small feeds are found most suitable.

IT is impossible to discuss all the points elucidate by these skiagraphic observations on the digestion of a bismuth feed, but reference may be made to the rate of travel through the intestine. A bismuth feed takes on an average about four hours to pass through the small intestine to the cæcum. About two hours are requ[illegible] to traverse the ascending colon, and the same time to pass the transverse colon to the splenic flexure. The subsequent course is more sluggish, and depends a good deal upon the previous contents of the lower bowels and the time of defæcation. Dr. Hertz used this method of investigation to examine cases of chronic constipation in order to ascertain what part of the bowel is responsible for the condition in any particular case. In two cases he found that the passage of food was normal as far as the transverse colon, and that the constipation was due to atony of the sigmoid and rectum, and in these cases daily enemata have relieved the condition. In other cases he has been able to locate the sluggish passage of food in the transverse colon, and in these cases he has obtained better relief by drugs, such as magnesium sulphate and liquor strychniæ. As Dr. Hertz sugests, this method opens up a wide field for research. Not only may it elucidate the pathology of constipation in the various conditions, neurasthenia, dyspepsia, chlorosis, with which it is associated, but it may also be possible by these means to discover the exact effect of the various drugs and other measures employed in the treatment of constipation, so that each case may be provided with a suitable treatment.

An interesting case of gangrenous kidney, diagnosed as perforated appendix and operated upon accordingly, is reported by Drs. Abernethy and Graham. The excised kidney was found to be enlarged and very black in colour. Its substance was very friable, and could be easily torn by pressure with the finger tips. No stone or pus could be found. The patient, a girl aged thirteen years, made a good recovery.

Moscati thinks that the presence or absence of glycogen in the placenta may be helpful, in medico-legal cases, towards arriving at a conclusion regarding the time of birth. All embryonic tissues are rich in glycogen and the placenta is especially so, containing on an average .58 centigram per 100 grammes of the freshly macerated organ. Under the action of the normal diastatic ferments this glycogen rapidly disappears; thus 20 minutes after its expulsion the percentage of glycogen in the placenta has decreased 50 per cent., and 23 hours after expulsion it is difficult to find even a mere trace of the carbo-hydrate in the placental tissues. Variations of temperature and the action of antiseptics to a certain extent modify this diastatic fermentation. Moscati's observations have not been confirmed, and the process of estimating the relative amounts of glycogen is too complicated for this method to be likely to come into general use.

At the last meeting of the Berlin Academy of Medicine, Dr. M. W. Körte showed a boy, aged 13, on whom he has operated several times for the cure of bronchiectasis, and who has had a large part of the right lung removed by open operation. The boy was admitted to the Hospital Urban in August 1906, with well-marked bronchiectatic cavitation in the right lower lobe. After the usual treatment, under which the boy got no better, it was decided to remove a section of rib and explore the chest. In this manner two large bronchiectatic cavities were evacuated, and the patient got slightly better. In May 1907, however, his condition was again very bad, and Gluck's operation was decided upon. This consists in the entire extirpation of one lobe, and was first practised as early as 1885. In this case the middle lobe was found most involved, and the greater part of it was accordingly removed. The boy made an excellent recovery. In the discussion that ensued the difficulties of the operation of resection of the lung were laid stress on. The hæmorrhage obscures the field, and the irritation of the pneumogastric nerves is exceedingly likely to cause collapse. Notwithstanding these difficulties the operation is becoming more popular with German surgeons, and Dr. Körte's splendid success will do much to encourage such popularity.

At a recent meeting of the Paris Obstetrical Society, M. Brindeau reported a case of an ovary, strangled within the sac of a hernia in a newly-born infant. At birth a large mass was observed in the inguinal region. This seemed to be acutely tender on palpation, and was irreducible. At the operation a gangrenous ovary was found in a hernial sac. The child recovered.

At the same meeting MM. Garipuy and Schreiber reported an interesting case, which gave rise to much discussion. A full-term child was extracted by means of forceps, delivery being quite easy, and the instrument being used merely to save time. Three days later the child became very ill, and died suddenly in a state of collapse. At the autopsy a large hæmatoma was found. This lay underneath the right lobe of the liver, and was due to a rupture of the suprarenal capsule. No other lesion could be found, and it was impossible to find out what had caused the rupture of the adrenal body. In the discussion that followed M. Bar stated that hæmorrhage into the substance of the suprarenal was by no means rare in newly-born infants. He thought it was always due to trauma, to syphilis, or to some primary infection, and they were always fatal.

Professor Cushny recently gave an address on nutmeg poisoning in the therapeutical and pharmacological section of the Royal Society of Medicine. He referred to the work of Dr. Wallace in America, who found that cases of poisoning occurred exclusively from the use of the crude nutmeg or mace. Although it has been used as an abortifacient, nutmeg does not appear to have this action. The symptoms are drowsiness, stupor, diplopia. Delirium is frequently present, and sometimes the first symptom is burning pain in the stomach, with præcordial anxiety or giddiness. The symptoms generally resemble those of cannabis indica. One fatal case occurred in a boy after eating two nutmegs. From experimental work Professor Cushny has come to the conclusion that the symptoms are to be attributed to action of the oil of nutmeg on the central nervous system. This is depressed; but there are some signs of stimulation in the form of restlessness, slight convulsive movements, and tremor. The oil has also a marked local irritant action, whether given by the mouth or hypodermically. The stomach wall is found red and injected, and the urine often contains albumen.

At the same meeting of the Royal Society of Medicine, Professor W. E. Dixon dealt with arterio-sclerosis and its causation. He referred to two theories of causation: (*a*) raising of blood pressure, and (*b*) toxic influences; and showed that it has been established by experiment that arterio-sclerosis can be produced in the lower animals by simply compressing the abdominal aorta for one minute once or twice a day for a comparatively short period. The changes consist in a drawing out of the elastic fibres and degeneration of the muscular coat, with the formation of placques of calcareous material. He explained how this might be brought about in man by violent exercise, more especially as age advances, since the rise of blood pressure after exertion is greater then than in youth. Dr. Dixon also spoke of the effect of nicotine on the blood pressure, comparing the sudden high rise in the beginner, after his first cigar, with the slight rise in the moderate smoker, and the absence of effect in the habitual smoker. He suggested that man produces a ferment, probably in the liver, which counteracts the effect of nicotine.

Some interesting experiments have been made in the Mahratta Plague Hospital, Bombay, by Dr. Choksy with the Yersinrout serum method of treatment in cases of plague. The mortality under ordinary treatment is about 87.13 per cent. on the average for the last four years. Dr. Choksy's records show that out of 200 cases treated by him with the serum there were 127 deaths and 73 recoveries, whilst among 200 cases in hospital who were not given this treatment there were 148 deaths and 52 recoveries.

Borget and Gengon, working at the Pasteur Institute at Paris, claim to have isolated the bacillus that is the cause of whooping cough. According to their latest paper the bacillus is only found in the first expectorations, which contain an almost pure culture of a small oval-shaped organism, which does not take the Gram stain. It may be grown upon gelatine plates or upon ascitic fluid, but the authors prefer a mixture of defibrinated blood, potato decoction and glycerin. No inoculation experiments have been tried, but the authors lay great stress on the fact that the serum of whooping cough patients only has an agglutinating effect upon cultures of the bacillus.

In a recent number of *La Normandie Médicale* Dr. Paul Brunon gives an account of his results with Chantemesse's serum in the treatment of typhoid fever. Injections of this serum, particularly when made during the first week, are followed by a sudden but transient reaction, characterised by marked elevation of temperature. This is rapidly (within two hours) followed by a period of defervescence, during which the temperature falls and the general state of the patient is markedly improved. Dr. Brunon believes that the serum treatment decidedly shortens the duration of the disease, and gives statistics in support of his contention. Before serum treatment was started, the mortality of his cases of typhoid fever was 17 per cent., but since he began using Chantemesse's serum it has dropped to 3 per cent.

Dr. Aly-Belfadel draws attention to an early sign of measles which is likely to be found extremely useful for diagnostic purposes. It is found in the limitation of the early conjunctivitis which is one of the first signs of the affection. In ordinary catarrhal conjunctivitis the affection begins and is most marked in the corners of the conjunctival sac, rarely affecting the bulbar reflection. In measles, however, the initial seat of the lesion is found to be on that portion of conjunctiva which is reflected over the eye-ball and to be most marked at the inner and outer side of the cornea (at the sites, in fact, of an ordinary pterygium). From this point the inflammation rapidly extends to include, when the rash has appeared, practically the whole of the conjunctiva. The author lays stress on this limitation which he has found constant in more than 70 per cent. of cases, and considers it a helpful diagnostic sign in early cases where the rash is not marked, and where no Koplik's spots are to be seen.

In a paper read before the French Academy of Medicine, M. Beclerc lays stress on the skiagraphic examination of all cases in which liver abscess is suspected. Such an examination not only accurately indicates the position of the abscess, and is therefore of great value if an operation is contemplated, but is perhaps the only means of confirming the diagnosis when the abscess is located between the diaphragm and the liver. As in the case of nephrolithiasis a screen examination is much more valuable than a photograph.

Picard in 1877, Paul Bert in 1878, Daries in 1883, and Gley and Richet in 1887, demonstrated that the minimal urinary secretion was during sleep and at night time. Le Faugeron, in a recently published thesis, claims that a consideration of the urinary excretion at night is of value in the diagnosis of lesions of the urinary tracts, and especially of the kidneys. His conclusions are that in cases where a definite kidney lesion exists, more urine is excreted while the patient lies down, and consequently the amount of day-urine is proportionately less than the amount excreted during the night.

Abe, who has investigated the *rôle* which animals play in the dissemination of enteric fever, reports the results of his experiments in a paper communicated to the Munich Academy of Medicine. In four cases of typhoid fever he found it was possible to demonstrate the presence of the typhoid bacillus in the bodies of lice taken from the persons and clothing of the affected patients. The insects were crushed in a sterile mortar, and cultures made from their bodies gave positive results. Thus a small portion of the crushed lice was injected under the skin of mice, with the result that the animals died of typhoid septicæmia. He repeated his experiments with fleas, taken from persons attending typhoid patients, but was unable to find any typhoid bacilli in these insects.

Russ, in an interesting paper, suggests the use of magnesium chloride as a substitute for plaster of Paris in permanent surgical dressings. Sorel, in 1869, called attention to the "setting" action of magnesium oxychloride when mixed with water, the resulting material being much harder, lighter and more capable of resisting wear than ordinary gypsum plasters. An additional advantage claimed by Russ is that the magnesium plasters are easily penetrated by the $x$-rays, whereas ordinary plaster is only penetrated with difficulty by the rays. The author uses commercial magnesite (which is practically an impure, or native, carbonate of magnesium), and a strong solution of magnesium chloride, and the technique of putting up a fracture is almost identical with that used in the case of ordinary plaster. As the cast can be made much thinner than with gypsum, the plaster is consequently much lighter. The only objection to this new material (and it seems a great objection) is that it takes much longer to set than does plaster of Paris. Once set, however, the plaster is practically imperishable, and may be polished.

